# Century of Milestones and Breakthroughs Related to the Immune Mechanisms of Atherosclerosis

**DOI:** 10.1161/ATVBAHA.124.319397

**Published:** 2024-04-25

**Authors:** Ziad Mallat, Alain Tedgui

**Affiliations:** 1 Department of Medicine, Section of CardioRespiratory Medicine, Victor Phillip Dahdaleh Heart and Lung Research Institute, University of Cambridge, United Kingdom (Z.M.).; 2 Institut National de la Santé et de la Recherche Médicale, Paris Cardiovascular Research Center, Université de Paris, France (Z.M., A.T.).

**Keywords:** adaptive immunity, atherosclerosis, cardiovascular diseases, innate immunity

After more than a century of atherosclerosis research, during which the wheel has been reinvented countless times, it is now widely accepted that atherosclerosis is a chronic inflammatory disease of large- and medium-sized arteries, initiated in response to the retention and accumulation of apoB-rich lipoproteins in the artery wall. Recent genome-wide association studies (GWASs) and large-scale phase 3 randomized clinical trials provided further support for the causal role of cholesterol and inflammation in atherosclerotic cardiovascular disease (ASCVD). While our arsenal of life-saving lipid-lowering therapies has grown considerably over the past decade, most anti-inflammatory drugs failed to make it to the clinic. Therefore, the need for safe and effective anti-inflammatory therapies in patients with, or at risk of, ASCVD remains unmet. Here, we will review major advances in the immune mechanisms of atherosclerosis over the past century (Figure) and provide a perspective on future research avenues that could lead to radically new strategies for prevention and treatment.

**Figure.**
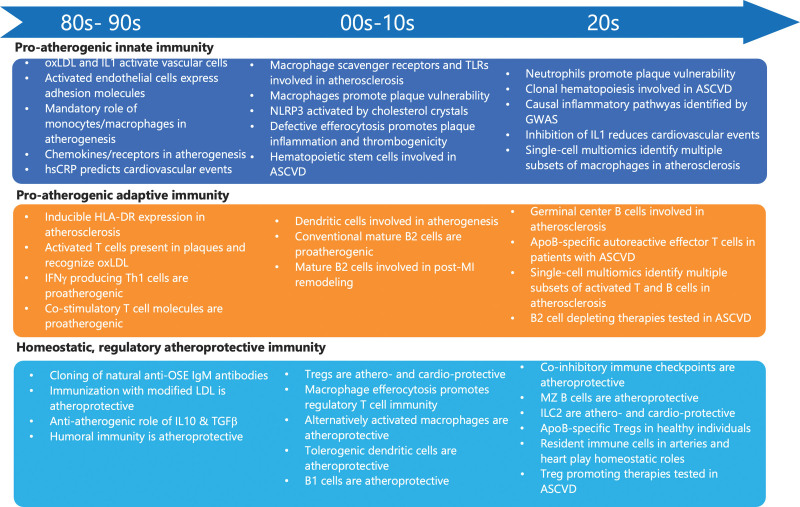
**Timeline of milestone events and breakthroughs related to innate and adaptive immunity in atherosclerotic cardiovascular disease (ASCVD).** GWAS indicates genome-wide association studies; HLA-DR, human leukocyte antigen-DR; hsCRP, high-sensitivity C-reactive protein; IL, interleukin; ILC2, type 2 innate lymphoid cell; LDL, low-density lipoprotein; MI, myocardial infarction; MZ, marginal zone; NLRP3, NOD-, LRR- and pyrin domain-containing protein 3; OSE, oxidation-specific epitope; oxLDL, oxidized low-density lipoprotein; TGFβ, transforming growth factor β; Th, T helper; TLR, toll-like receptor; and Treg, regulatory T.

## INNATE IMMUNITY

Remarkably, the role of inflammation in the pathogenesis of atherosclerosis was recognized early on with the term chronic endarteritis deformans coined by Virchow in 1856 and the description by Anitschkov in 1913 of fat-filled macrophages in atherosclerotic plaques of rabbits fed a cholesterol-rich diet. Renewed interest in the role of macrophages in atherosclerosis resurfaced several decades later from the late 1950s to the early 1980s, particularly with the work of Poole/Florey and Gerrity/Schwartz, describing the adherence and migration of blood monocytes through an intact endothelium in response to cholesterol feeding, promoted by chemoattractants secreted by smooth muscle cells, along with their differentiation into fat-laden macrophages within intimal fatty streaks.

In the 1980s, work by Steinberg/Witztum, Chisolm, and Berliner/Fogelman pointed to a potentially important role of (oxidative) modifications of low-density lipoprotein that increases its atherogenicity, with 1 potential initiating mechanism being the stimulation of monocyte endothelial interactions,^1^ which was later attributed to the induction of adhesion molecules on endothelial cells and their interactions with activated integrins on monocytes thanks to work by Cybulsky and Gimbrone.^2^ The causal role of inflammation was then tested using genetically modified mice, which established an obligatory role of monocytes/macrophages in disease development,^3^ as well as a causal role for defined chemokine-chemokine receptor and adhesion molecule-integrin pathways. Subsequent work defined the role of macrophages within the plaques. The first receptor for oxidized low-density lipoprotein (oxLDL) was identified by Endemann in 1993, followed by work by Moore/Freeman and Febbraio/Silverstein defining the role of CD36 in foam cell formation during atherosclerosis, and later extending its role to sterile inflammatory activation of macrophages in concert with toll-like receptors, as well as its role in coordinating NLRP3 (NOD-, LRR- and pyrin domain-containing protein 3) inflammasome activation by facilitating intracellular nucleation of oxLDL into crystals. Along with a role of macrophages in plaque development, studies by Davies, Virmani, Henney, Galis/Libby, Falk, Shah, and Badimon/Fuster established critical roles for macrophages in plaque disruption leading to acute ischemic events, in particular through inhibition of collagen synthesis, production of matrix metalloproteinases within the fibrous cap, and promotion of plaque thrombogenicity through increased expression and activation of tissue factor.

The accumulating evidence on the inflammatory hypothesis of atherosclerosis instigated by lipoprotein retention and modifications led Ross to revisit his response to injury hypothesis, which initially postulated a prominent role of platelet activation and PDGF (platelet-derived growth factor)-dependent smooth muscle cell proliferation following endothelial injury. The finding by Ross and others that PDGF can also be produced by plaque macrophages and inflammatory mediators are involved in modulating smooth muscle cell phenotype was compatible both with the lipid retention and inflammation hypotheses and with the smooth muscle cell phenotypic modulation hypothesis originally proposed by Wissler and later developed by Ross and Campbells. This led to the wide recognition and acceptance of atherosclerosis as a chronic inflammatory disease.^4^ The importance of inflammation was further supported by the strong association of CRP (C-reactive protein), a systemic marker of inflammation, with future cardiovascular events.^5^

As in other chronic inflammatory diseases, the perpetuation of inflammation in atherosclerosis has been linked to defective inflammation resolution, including work by Tabas on defective efferocytosis of apoptotic cells, and defective production of specialized proresolving mediators. Resolution of inflammation has been shown to be essential for plaque stabilization or regression. These findings are being harnessed for potential therapeutic use, as is the case with CD47-blocking antibodies that promote efferocytosis. The recent finding that icosapent ethyl reduced cardiovascular events also suggested possible anti-inflammatory effects through enhanced generation of specialized proresolving mediators. However, no such evidence has been provided to date.

The most impactful research on the role of inflammation in ASCVD came from work initiated in the 1980s by Loppnow and Libby^6^ on the role of the IL (interleukin)-1/IL-6 pathway. They showed that vascular cells responded to IL-1 by producing an array of inflammatory cytokines, including IL-1 itself and IL-6.^6^ Two decades later, the discovery of the inflammasomes, and in particular that NLRP3 inflammasome activation by cholesterol crystals promotes atherosclerosis through IL-1 activation,^7^ accelerated efforts to translate this concept to the clinic. This led to the CANTOS trial (The Canakinumab Antiinflammatory Thrombosis Outcome Study) that showed significant reduction of cardiovascular events after IL-1β neutralization with canakinumab in patients with previous myocardial infarction and elevated high-sensitivity CRP levels.^8^ However, treatment with canakinumab was associated with an increased risk of fatal infections. Subsequently, treatment with colchicine, a drug previously shown to inhibit NLRP3, has been associated with a significant reduction of ischemic cardiovascular events.^9^ Many other specific NLRP3 inhibitors and inhibitors of other inflammasomes, which are shown to be involved in atherosclerosis, are in development.

A breakthrough in our understanding of the causal mechanisms of human ASCVD came from GWAS, later combined with the study of multiomic traits. One of the most reproducible hits relates to the IL-6 pathway,^10^ which led to the development of several therapies to inhibit IL-6 signaling. The outcomes of these trials will be available in the next few years.

More recent studies further highlighted the role of the innate immune system in mediating ASCVD. New mechanisms include the role of trained immunity in ASCVD, the role of medullary and extramedullary hematopoiesis in general and clonal hematopoiesis in particular,^11^ new roles for neutrophils in plaque development and disruption, the role of the gut microflora, the impact of lifestyle factors and environmental and social stress on myelopoiesis through neuroimmune mechanisms,^12^ and the recent description of neuroimmune cardiovascular interfaces that control atherosclerosis. The years ahead will tell whether and how these mechanisms can be exploited for better prevention and treatment of patients with ASCVD.

## PROATHEROGENIC ADAPTIVE T-CELL IMMUNITY

Formal identification of T cells in atherosclerotic lesions was provided by Jonasson et al^13^ in the early 1980s along with signs suggestive of HLA-DR (human leukocyte antigen-DR)–driven adaptive immunity. The finding by Pober that class II histocompatibility antigens can be induced on vascular cells by T-cell–derived IFNγ (interferon gamma) further supported the possibility of a local adaptive T-cell activation. Then, T- and B-cell–deficient mice were found to have reduced atherogenesis under moderate hypercholesterolemia,^14^ which was reversible by transfer of CD4+ T cells, establishing a proatherogenic role for adaptive T-cell immunity. This was further extended by others showing a proatherogenic role of costimulatory T-cell molecules and IFNγ-producing type 1 T helper cells.

In 1989, Witztum and colleagues identified anti-oxLDL antibodies and showed that they were associated with atherosclerosis progression. Wick and colleagues also showed an association between anti-HSP65 antibodies and carotid atherosclerosis. Soon thereafter, Stemme et al^15^ showed that T cells isolated from atherosclerotic lesions responded to oxLDL in an HLA-DR–dependent manner. This was further extended by the discovery of several other atherosclerosis-related autoantigens and the finding of a strong association of autoimmune diseases with ASCVD. Autoantigens are presented by specific subsets of dendritic cells to activate T cells. Intensive research by Ley and others is under way to characterize autoantigens and their specific T cells in patients with ASCVD.

## ATHEROPROTECTIVE B-CELL HUMORAL IMMUNITY

In the early 1990s, Palinski et al^16^ made the surprising observation that immunization with oxLDL was atheroprotective, which was soon confirmed by Nilsson et al. They cloned monoclonal autoantibodies to oxidation-specific epitopes (OSEs) and showed that anti-OSE IgM autoantibodies shared identity with other natural antibodies.^17^ This sparked intensive research into the atheroprotective role of anti-OSE antibodies supported by the finding that total B-cell deficiency accelerates atherosclerosis and supplementation of splenectomized mice with splenic B cells reduces disease development. Subsequent studies attributed the production of atheroprotective anti-OSE IgM antibodies to innate B1 cells and, more recently, to marginal zone B cells. The strong inverse association of anti-OSE IgM antibodies with ASCVD and the anti-inflammatory and atheroprotective effects of OSE neutralization^18^ suggest a potential therapeutic strategy.

## COUNTERREGULATORY IMMUNE MECHANISMS

By the end of the 1990s, it was generally admitted that T cells were proatherogenic, whereas B cells were atheroprotective. Yet, immunologists had identified subsets of immune cells with counterregulatory properties, including classically versus alternatively activated macrophages, and IL-10–producing T cells that suppress type 1 T helper cells. That led us to reconsider the role of immune cells and cytokines in atherosclerosis. Our laboratory first showed that the anti-inflammatory cytokines IL-10 and TGFβ (transforming growth factor β) were atheroprotective followed by the discovery of an essential role of regulatory T cells in atheroprotection.^19^ Following the same principle of counterregulatory immune responses, we searched for proatherogenic B-cell subsets that may oppose the atheroprotective role of innate B1 cells. We found, as well as others, that mature B2 cells expressing CD20 carried proatherogenic immunity^20^ and played a detrimental role in cardiac remodeling after myocardial infarction. These preclinical findings are now being translated into the clinic, with the use of low-dose IL-2 to expand regulatory T cells and the use of a monoclonal anti-CD20 antibody, rituximab, to deplete mature B2 cells in patients with acute myocardial infarction.

The concept of a critical role of counterregulatory immune pathways in atherosclerosis was more recently extended to other immune cell types, including regulatory and IL-10–producing B cells and innate lymphoid cells type 2, and to inhibitory atheroprotective immune checkpoints by opposition to costimulatory proatherogenic checkpoints.

## PERSPECTIVE ON FUTURE RESEARCH AVENUES

Despite these important advances, many anti-inflammatory therapies were lost in translation, calling into question the relevance of preclinical models for human disease. While preclinical models are useful for establishing general concepts (eg, cholesterol and inflammation play causal roles in atherogenesis), more specific pathways should be studied directly in humans to accelerate translation. This task is now greatly facilitated by the advent of GWAS, coupled with multiomics and multimodality molecular imaging of atherosclerotic plaques, which enable the delineation of causal molecular pathways with great precision at single-cell resolution. These technologies will revolutionize our understanding of the immune mechanisms of ASCVD and will play a decisive role in characterizing antigen-specific adaptive immune responses, paving the way for selective immunomodulatory therapies.

We are also focused on the advanced stages of ASCVD, whereas GWAS data integrate lifelong exposure to potentially causative pathways. Contrary to current assumptions, those pathways may not be constantly mobilized throughout life, which may have major implications for the timing of a specific intervention. If a causal pathway, identified through GWAS, is predominantly active during childhood and the early stages of plaque development, targeting this pathway later in life in patients with advanced ASCVD may not be effective. Other inflammatory pathways may be adaptive at some stages of plaque development but maladaptive at other stages. Studying ASCVD across the life span will be essential to identify trajectories of health and disease, as well as timepoints where these trajectories diverge, paving the way for personalized, effective, and safe preventive therapies.

Finally, we should make a paradigm shift in the way we consider the role of the immune system in ASCVD. At present, immune cells are mainly considered in terms of their contribution to disease, but we should place greater emphasis on the role of immune cells in homeostasis and recognize that resident immune cells, including subsets of macrophages, regulatory T cells, type 2 innate lymphoid cells, etc, are an integral part of the cardiovascular tissue, with their neuroimmune cardiovascular interactions playing essential homeostatic roles. Therefore, restoring the number and function of resident immune cells after tissue injury will be essential to restore tissue repair and enhance tissue tolerance to disease, beyond their anti-inflammatory properties.

### Note

We have barely scratched the surface of the extensive body of research about the role of the immune system in ASCVD. We are frustrated and apologize to the researchers and teams whose work could not be cited or acknowledged appropriately within the limited space allotted to us.

## ARTICLE INFORMATION

### Sources of Funding

British Heart Foundation and INSERM.

### Disclosures

None.
